# Development of Rapid Disk Diffusion Device Using Laser Speckle Formation Technology for Rapid Antimicrobial Susceptibility Testing

**DOI:** 10.1007/s00284-024-03798-3

**Published:** 2024-07-14

**Authors:** Jaehyeon Lee, Jun Han Lee, Kyoungman Cho, Jeong Su Park

**Affiliations:** 1 Department of Laboratory Medicine, Jeonbuk National University Medical School and Hospital, Jeonju, Republic of Korea; 2 Research Institute of Clinical Medicine of Jeonbuk National University-Biomedical Research Institute of Jeonbuk National University Hospital, Jeonju, Republic of Korea; 3 The Wave Talk., Inc., Jinri Hall, 193, Munji-Ro, Yueseong-Gu, Daejeon, 34051 Republic of Korea; 4 Department of Laboratory Medicine, Seoul National University Bundang Hospital, Seoul National University College of Medicine, Seongnam, Republic of Korea

## Abstract

The escalation of antimicrobial resistance (AMR) due to the excessive and inappropriate use of antimicrobials has prompted the urgent need for more rapid and effective antimicrobial susceptibility testing (AST) methods. Conventional AST techniques often take 16–24 h, leading to empirical prescription practices and the potential emergence of AMR. The study aimed to develop a rapid disk diffusion (RDD) method utilizing laser speckle formation (LSF) technology to expedite AST results. The study aimed to evaluate the performance of LSF technology in determining antimicrobial susceptibility. In this study, preclinical and clinical settings were established to compare the LSF technology with conventional disk diffusion (DD) methods to measure the inhibition zones. Preclinical experiments with different bacterial strains demonstrated more than 70% categorical agreement (CA) against most antimicrobials. Further, clinical experiments with multiple strains and antibiotics revealed CA ranging from 40 to 79%, while major and minor discrepancies were observed around 30% and 11%, respectively. These observations revealed high concordance between RDD and DD for multiple antimicrobials in multiple species. The results underscore the potential of RDD-based LSF technology for hastening AST procedures. The current study is marked by a unique equipment setup and analysis approach. Collectively, the suggested laser-based RDD showed greater potential than previously developed comparable methods. The proposed method and design have a higher application potential than formerly developed similar technologies. Together, the study contributes to the ongoing development of rapid AST methods.

**Supplementary Information:**

The online version contains supplementary material available at 10.1007/s00284-024-03798-3.

## Introduction

Antimicrobial resistance (AMR) is an increasingly prevalent global concern that is constraining the efficacy of antibiotics in the treatment of infections. Antibiotics is vital in treating bacterial, viral, fungal, or parasitic infections. Antimicrobials are specifically designed to eliminate bacterial pathogens, thereby preventing the spread of infectious diseases and improving overall public health. However, the overuse and misuse of antimicrobials have led to an increase in the development of antimicrobial resistance, which reduces the effectiveness of antimicrobials and poses a major threat to healthcare globally. Antimicrobial susceptibility testing (AST) is essential in preventing the misuse of antimicrobials and the development of resistance [1]. It is a routine clinical procedure that determines the susceptibility of a pathogen to a specific antimicrobial and its concentration. AST provides information on the efficacy of a particular antimicrobial, assisting physicians in selecting the ideal treatment for their patients. However, manual AST methods are labor intensive and time consuming. Moreover, AST require a high level of expertise, and any oversight in analysis can easily result in human errors [2]. Specifically, acquiring the desired information on antimicrobials using standard AST methods takes more than 16–24 h due to the lengthy procedure involved in culturing the bacteria. Solid-based ASTs, such as agar dilution, disk diffusion, and E-test, often need a minimum of 16–24 h as they demand the visual growth of bacterial colonies. Similarly, liquid-based ASTs, such as broth microdilution or the use of automated systems like VITEK system or Phoenix system that measure the optical density of bacterial solutions, assess bacterial growth under specific antimicrobial conditions. However, it takes 16 to 24 h to produce the expected outcome [3, 4]. Thus, the turnaround time associated with these ASTs presents challenges for physicians in their decision-making process for selecting an initial treatment. This often forces physicians to recommend initial antimicrobials based on clinical judgement, which carries the risk of prescribing antimicrobials and their concentrations that are inappropriate, potentially promoting AMR. Therefore, there is an urgent need for more rapid ASTs that can offer initial guidance to physicians to prevent the AMR crisis.

In search of faster ASTs, multiple diagnostic techniques have been developed over the decades to achieve rapid AST. Several approaches to enhance AST have been developed, including genomic tests, biosensors, isotype labeling, and hybridization techniques [5–8], but most of these techniques are yet to be standardized. Most modern methods rely on high-resolution imaging and time-lapse surveys performed at multiple locations, making them expensive for high-volume operations. Besides, some rapid methods used Raman spectroscopic biomarkers, DNA probes, and RNA markers to investigate antimicrobial susceptibility patterns. However, their broad applicability for multiple strains and antibiotics remains debatable. Disk diffusion (DD) is a traditional approach to determining bacterial susceptibility to antimicrobial agents. Despite being a traditional approach, it continues to be widely used in hospital laboratories due to its straightforwardness and the ease with which specific antimicrobials can be chosen for examination. However, this method needs 16 to 24 h to complete the task, which presents a time obstacle for physician in their treatment decision-making process and becomes a significant drawback of the approach. The European Committee on Antimicrobial Susceptibility Testing (EUCAST) has developed a rapid disk diffusion method that uses positive blood culture as the inoculum for a standard DD test, which can provide results in 4 to 8 or 16 to 18 h [9]. However, this technique is restricted to specific microbes and antimicrobials. The proposed advantages of implementing a rapid DD technique for multiple antimicrobials across a broader spectrum of bacterial species include expediting evidence-based dosage adjustment for antimicrobials. This would enhance treatment efficacy and impede the emergence of AMR. Furthermore, previous investigations on using laser speckle time series images for the dynamic visualization of inhibition zones by antimicrobials demonstrated the ability to detect these areas earlier than conventional approaches [10]. In these investigations, laser was employed to illuminate the sample dish and capture the speckle pattern that is reflected from the surface of the dish for measurements. Here, capturing the reflected image necessitates detecting the position and size of antibiotic disks over time through correlation calculations. However, due to variations in the reflective patterns caused by different antibiotic disks, precise determination becomes challenging. Hence, a unique approach is required to overcome the obstacles associated with this approach. Moreover, use of algorithms may exhibit limitations in identifying rare medical conditions as these methods are typically trained on extensive datasets that predominantly consist of instances of common conditions rather than rare ones [11]. Thus, using algorithms might result in data biases and ethical considerations [12, 13]. A recent study showed that implementing a rapid microbiological protocol for detecting bloodstream infections offered favorable results [14]. These investigations promote the utilization of Light Scattering Technology (LST) for expedited diagnosis. In this study, a validated laser speckle formation (LSF) technology [15–18] was applied to calculate the area inhibited by the antimicrobials by comparing the image of the initial sample prepared by the conventional DD method with the image of the sample after 4 h. Specifically, performance of a rapid disk diffusion (RDD) method based on LSF technology was evaluated by comparing the susceptibility of each antimicrobial agent determined by the RDD method with that determined by the conventional DD method for representative Gram-positive and Gram-negative bacteria.

## Methods

### Instruments and Hardware

**Fig. 1:**
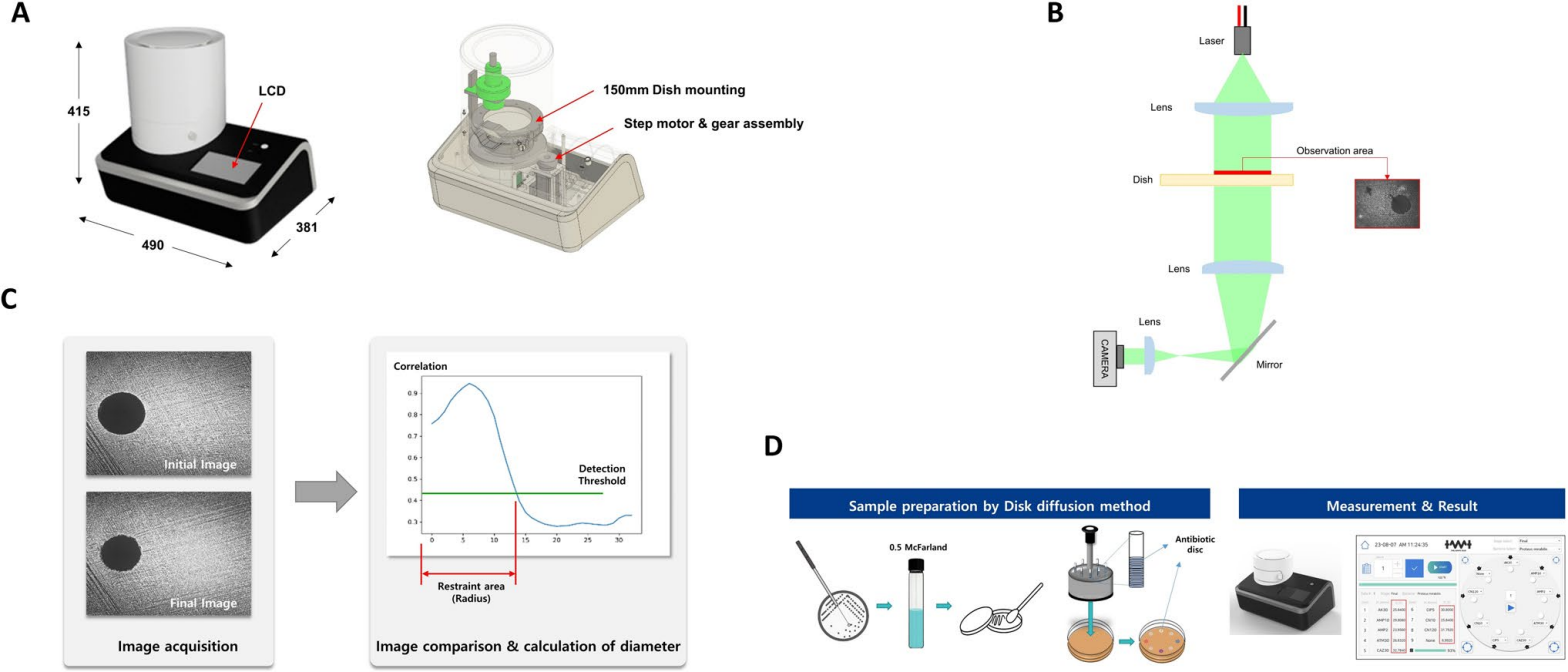
Illustrations on the device and their working principle. **A** Laser scattering-based antimicrobial susceptibility test (LS-AST) instrumentation and hardware for image analysis. LS-AST instrument is a bench-top device, measuring 490 mm in width, 415 mm in length, and 415 mm in height. It features a touch LCD for control and a front tray designed to accommodate a 150 mm petri dish for measurements, along with a stepper motor and gears installed to facilitate the rotation of the mounted dish. **B** Optical design of LS-AST instrument. The instrument has a lens to make sure that the light from the source (Laser 532 nm) stays parallel, and a polarizer is installed to create a uniform polarization state. This design choice prevents the instrument's height from becoming excessively high while maintaining the necessary optical characteristics without compromising its performance. **C** Measurement principle of LS-AST image analysis. **D** Schematic diagram showing representative work flow to perform disk diffusion method. Well-isolated colonies were cultured on a tryptic soy agar (TSA) plate. Then, a 0.5 McFarland suspension was prepared from overnight cultures. Using a swab, the organism was inoculated onto a Mueller–Hinton agar (MHA), and corresponding antimicrobial disks were applied. Next, initial and secondary measurements were taken in LS-AST device. The inhibition zone was estimated by comparing these results. Later, plates were cultured for up to 18 h to assess inhibition zones using reflected light

The Laser scattering-based antimicrobial susceptibility test (LS-AST) instrument is a bench-top device, measuring 490 mm (W) × 381 mm (L) × 415 mm (H), and has a weight of 13 kg, equipped with a touch LCD to control input and output (I/O) devices. Also, a front tray is specifically designed to accommodate a 150 mm petri dish for measurements. Besides, a stepper motor and gears are installed to facilitate the rotation of the mounted dish, ensuring an easy and efficient power transmission. Moreover, there is a single USB 3.1 port for the transfer of stored data to an external storage device. Figure 1A shows the representative image of the instrument. The Bacometer device was used to acquire high-resolution images of bacterial cultures at present time intervals. Subsequently, these images were processed and analysed to measure the extent of bacterial growth and ascertain the sensitivity of the bacteria to antibiotics. The protocol ensures uniform lighting conditions, focus, and magnification settings that maintain precision and reliability.

### Optical Design

Optical design is a crucial aspect of instrument development, focusing on manipulating light for optimal performance. A lens is installed to ensure that the light from the light source (Laser 532 nm) is kept parallel and has a light irradiation area over the observation area. Additionally, a polarizer is installed to create a uniform polarization state. The distance from the sample to the sensor was approximately 250 mm, depending on the focal length to meet the specified magnification. Further, a mirror was inserted to fold the optical path, forming a horizontal width, and preventing the instrument's height from becoming excessively high (Fig. 1B). This novel design maintains the optical characteristics without compromising on its optical performance.

### Measurement Principle

Agar is a transparent medium. Therefore, a parallel wavefront is produced when a laser with a parallel wavefront passes through it. However, in the presence of a colony, scattering occurs when a laser with a parallel wavefront passes through it. This scattered light and the wavefront interfere with the laser with a parallel wavefront, producing a narrow rice-like pattern. By analyzing this pattern, the growth of bacteria, which is invisible to the eye, can be confirmed. The spatial correlation coefficient was used to quantify the bacterial growth and non-growth areas. Subsequently, the diameter of the inhibition zone is determined by comparing the image of the initial sample obtained using the conventional DD method with the image of the sample at the time when the signal due to the bacterial colony appears. Still, positional fluctuations between the initial and subsequent image acquisitions are possible. Thus, comparisons must be made within the same spatial coordinates. To overcome the positional fluctuations and improve accuracy, a reference position, such as an antimicrobial disk, needs to be specified to calibrate the different coordinates. In addition, if there are more than two antimicrobials in a single petri dish, a relationship expression for the position and angle of the antimicrobial disk can be obtained to correct the position of the misaligned image automatically. Figure 1C illustrates the measurement principle of LS-AST.

## Preclinical Experiments

### Bacterial Strains

This study used well-characterized reference strains for preclinical investigations, ensuring consistency and accuracy in evaluating inhibition zones. Also, using these strains enhances the validity and reproducibility of research findings in AST. The experiment comprised a total of 44 strains,, including 11 strains each of *Escherichia coli, Pseudomonas aeruginosa, Enterococcus faecalis,* and *StaphylococcuS. aureus.* Multiple strains of *Escherichia coli* were obtained from ATCC (American Type Culture Collection) and CCARM (Culture Collection of Antimicrobial Resistant Microbes, Republic of Korea). The specific strains of *E. coli* obtained were ATCC 25922, CCARM 1A050, CCARM 1A076, CCARM 1A525, CCARM 1B669, CCARM 1B695, CCARM 1G109, CCARM 1G490, CCARM 1G517, NCCP (National Culture Collection for Pathogens, Republic of Korea) 14,629, and NCCP 16283. Similarly, multiple strains of *Pseudomonas aeruginosa* were obtained from ATCC and CCAR. The specific strains of *P. aeruginosa* obtained were ATCC 27853, CCARM 2092, CCARM 2095, CCARM 2244, CCARM 2271, CCARM 2191, CCARM 2198, CCARM 2206, CCARM 2235, CCARM 2282, and CCARM 2325. The specific strains of *Enterococcus faecalis* obtained were ATCC 29212, CCARM 5115, CCARM 5172, CCARM 5185, CCARM 5204, CCARM 5466, CCARM 5515, CCARM 5531, CCARM 5543, CCARM 5555, and CCARM 5560. Likewise, multiple strains of *StaphylococcuS. aureus*, such as ATCC 25923, ATCC 29213, CCARM 3795, CCARM 3799, CCARM 3800, CCARM 3807, CCARM 3808, CCARM 3816 2325, NCCP 14558, NCCP 14565, and NCCP 15869 were obtained from TCC, CCARM, and NCCP.

### Methods

The DD method was carried out in accordance with the CLSI M02 guidelines [19]. Briefly, three to five well-isolated colonies of the test organism were cultured on a tryptic soy agar (TSA, Bacto™, BD, USA) plate. Next, a suspension equivalent to a 0.5 McFarland standard was prepared in 0.85% saline prepared from overnight cultures. Using a swab, the organism was inoculated onto a Mueller–Hinton agar (MHA, Asan Pharmaceutical, Hwaseong, Korea) plate. For each MHA plate, corresponding antimicrobial disks were applied. Disks are positioned equidistantly on the agar surface to achieve uniform exposure of microbes to antibiotics. The arrangement adheres to standardized guidelines, such as those provided by the Clinical and Laboratory Standards Institute (CLSI), to ensure that the arrangement remains consistent and enables reproducibility between tests. Disk spacing is meticulously planned in order to avoid overlap and assure the presence of distinct inhibition zones for each antibiotic. To perform RDD, the plates were put in the LS-AST device, and the initial measurement was taken. Then plates were incubated for 4 h at 35 °C, and the secondary measurement was taken on an LS-AST device. The inhibition zone was estimated by comparing the results of the initial and secondary measurements. The plates were then put back into the incubator and incubated for up to 16 to 18 h at 35 °C to perform the final manual measurement. Specifically, the initial and second measurements were merged with the final measurement to forecast the projected bacterial inhibition values produced by the equipment. These observations were used to compare the visual assessment. Schematic diagram of the work flow is shown in Fig. 1D.

The disks meant for *E. coli* contain different concentrations of different antimicrobials. The *E. coli* disks contain the following concentrations of antimicrobials. 300 μg of nitrofurantoin, 100 μg of nitrofurantoin; 30 μg of amikacin, aztreonam, chloramphenicol, ceftazidime, cefotaxime, cefepime, cefoxitin, and cefazolin; 20/10 μg of amoxicillin-clavulanic acid; 10 μg of ampicillin, gentamicin, imipenem; 5 μg of ciprofloxacin; 10/10 μg of ampicillin-sulbactam; 1.25/23.75 μg of trimethoprim-sulfamethoxazole. Similarly, The *P. aeruginosa* disks contain varying amounts of distinct antimicrobials. The *P. aeruginosa* disks contain the following concentrations of antimicrobials. 100/10 μg of piperacillin-tazobactam; 30 of μg amikacin, aztreonam, ceftazidime, cefepime; 10 μg of gentamicin, imipenem, and tobramycin; 5 μg of ciprofloxacin, levofloxacin. Likewise, *E. faecalis* disks contain the following concentrations of antimicrobials. 300 μg of nitrofurantoin and streptomycin; 100 μg of nitrofurantoin; 120 μg of gentamicin; 30 μg of linezolid and vancomycin; 10 μg of ampicillin and norfloxacin; 5 μg of ciprofloxacin, levofloxacin, and vancomycin. Similarly, *S. aurues* disks contain the following concentrations of antimicrobials. 30 μg of cefoxitin, linezolid, and tetracycline; 15 μg of erythromycin; 10 μg of gentamicin; 5 μg of rifampin; 2 μg of clindamycin; 0.01 μg of penicillin; 1.25/23.75 μg of trimethoprim-sulfamethoxazole. DD results were evaluated using 2022 CLSI disk breakpoints [20].

## Clinical Experiment

### Strain Selection

The preclinical observations were validated with blood and respiratory cultures collected between 2020 and 2022 from Seoul National University Bundang Hospital, Korea and Jeonbuk National University Hospital, Jeonju, Korea. Gram-positive bacteria like *S. aureus* and *E. faecalis* and gram-negative bacteria like *Klebsiella pneumoniae, Proteus mirabilis,* and *P. aeruginosa* were used for the clinical experiments. All the collected gram-negative and gram-positive samples were cultures in blood agar and MacConkey agar plates under standard growing conditions for up to 24 h and identified using the MicroScan (Siemens Healthcare Diagnostics, Deerfield, IL) and Vitek MS system (BioMérieux, Hazelwood, MI, USA), respectively. Further, the Vitek 2 AST 211 cards (BioMérieux, Marcy-l’Étoile, France) were used to carry out ASTs and interpreted with the VITEK 2 identification system. The selected microorganisms, such as *S. aureus*, *E. faecalis*, *K pneumoniae*, *P mirabilis*, and *P. aeruginosa* are often linked to multiple infections and are frequently encountered in clinical settings. This enables a comprehensive assessment of the effectiveness of antimicrobial agents in various types of illnesses. All the clinical isolates were stored in skimmed milk at ≤ 70 °C until use.

### Methods

DD was performed by following the CLSI M02 guidelines [19]. The *S. aureus* disks contained the following amounts of antimicrobials. 30 μg of cefoxitin, linezolid, and tetracycline; 15 μg of erythromycin; 10 μg of gentamicin; 5 μg of ciprofloxacin, rifampin; 2 μg of clindamycin; 1.25/23.75 μg of trimethoprim-sulfamethoxazole. Likewise, the *E. faecalis* disks contained the following amounts of antimicrobials. 300 μg of nitrofurantoin and streptomycin; 120 μg of gentamicin; 30 μg of linezolid, teicoplanin, and vancomycin; 10 μg of ampicillin. Similarly, *K. pneumoniae* disks contained the following amounts of antimicrobials. 30 μg of ceftazidime, cefotaxime, cefepime, cefoxitin, and cefazolin); 10 μg of gentamicin and imipenem; 5 μg of ciprofloxacin; 10/10 μg of ampicillin-sulbactam. *P. mirabilis* disks contained the following amounts of antimicrobials. 30 μg of amikacin, cefotaxime, cefepime, and cefoxitin; 10 μg of ampicillin, gentamicin, imipenem, and tobramycin; 5 μg of levofloxacin. *P. aeruginosa* disks contained the following amounts of antimicrobials. 30 μg of amikacin, aztreonam, ceftazidime, and cefepime; 10 μg of imipenem and tobramycin; 5 μg of ciprofloxacin, levofloxacin; 100/10 μg of piperacillin-tazobactam.

### Data Analysis

The interpretative results were categorized as susceptible (S), intermediate (I), and resistant (R). All the categorizations from DD manual measurements and RDD with LS-AST device image analysis were performed. Further, categorical agreements (CA) and discrepancies were determined. The CA of susceptibility was calculated using the DD measurement as the reference observation. The CA for various antimicrobials in different strains was compared between DD measures and RDD with LS-AST using Fisher's exact test (Table 1). The statistical calculations were performed using a two-tailed method, and a *P*-value < 0.05 was considered statistically significant. A *P*-value < 0.05 illustrates how the two approaches diverge in their observations of susceptibility categorization. Discrepancies were recorded as major discrepancy (MD) when DD with manual measurement indicated resistance, and the RDD indicated susceptibility and vice versa. Minor discrepancies (mD) were recorded when DD with manual measurement indicated intermediate, and the RDD indicated susceptibility or resistance or when DD with manual measurement indicated susceptibility or resistance and the RDD indicated intermediate. In the tests, high CA indicates that the test reliably predicts whether a particular antimicrobial will be effective in treating an infection caused by the organism tested. Otherwise, MD can lead to inappropriate therapy, potentially resulting in treatment failure and worsening of the patient's condition. The acceptable level of CA can vary slightly depending on the regulatory guidelines, the specific organism-antibiotic combinations being tested, and the clinical setting in which the AST is being tested. In this study, CA with a 90% or higher threshold was used to ascertain the method's appropriateness for clinical application. In addition, we computed standard metrics to assess the effectiveness of RDD, including sensitivity, specificity, positive predictive value (PPV), negative predictive value (NPV), accuracy, positive likelihood ratio (PLR), negative likelihood ratio (NLR), and diagnostic odds ratio (DOR). Sensitivity refers to the proportion of bacteria that are susceptible and yield a susceptible result (S), while specificity refers to the proportion of bacteria that are not susceptible and yield a non-susceptible result ( I or R).

**Table 1:** Comparative performance of disk diffusion with manual inhibition zone measurement and rapid disk diffusion test

2Strain	Antimicrobials	Manual method			LS-rapid disk diffusion (LS-AST method)			CA (%)	MD (*n*)	mD (*n*)	Fisher’s exact test *P*-value
		S	I	R	S	I	R				
*S. aureus*	Ciprofloxacin	130	0	22	126	4	22	77.6	30	4	0.0269
	Gentamicin	122	1	29	110	0	42	78.3	32	1	0.0001
	Clindamycin	146	0	6	124	0	28	78.9	32	0	1.0000
	Erythromycin	113	0	39	113	4	35	68.4	44	4	0.0052
	Cefoxitin	98	0	54	71	4	77	50.7	71	4	0.7355
	Linezolid	152	0	0	116	0	36	76.3	36	0	1.0000
	Rifampin	152	0	0	119	0	33	78.3	33	0	1.0000
	Trimethoprim/sulfam	152	0	0	116	0	36	76.3	36	0	1.0000
	Tetracycline	148	0	3	120	1	30	78.1	32	1	0.5007
***E. faecalis***	Ampicillin	143	0	11	104	0	50	68.2	49	0	0.1773
	Nitrofurantoin	154	0	0	92	0	62	59.7	62	0	1.0000
	Linezolid	144	8	2	90	0	64	53.2	64	8	0.1959
	Teicoplanin	153	1	0	94	2	58	60.4	58	3	1.0000
	Vancomycin	153	0	1	95	1	58	61	59	1	1.0000
	Gentamicin HL	116	0	38	86	22	46	52.6	51	22	0.1335
	Streptomycin HL	148	0	6	90	26	38	61	34	26	0.0045
***Klebsiella pneumoniae***	Ceftazidime	58	0	59	28	0	89	53.0	47.0	0	0.3927
	Ciprofloxacin	36	12	69	9	19	89	57.3	17.9	31	0.0036
	Gentamicin	79	1	37	71	8	38	63.2	29.1	9	0.0166
	Cefotaxime	46	2	69	20	27	70	51.3	23.9	29	0.3205
	Cefepime	48	8	61	24	10	83	57.3	29.1	18	0.0231
	Cefoxitin	84	6	27	24	15	78	36.8	48.7	21	0.4519
	Imipenem	97	2	18	49	23	45	42.7	35.9	25	0.8012
	Cefazolin	32	10	75	43	16	58	43.6	41.9	26	0.0528
	Ampicillin-sulbactam	34	13	70	29	21	67	44.4	29.9	34	0.3466
***Pseudomonas aeruginosa***	Amikacin	63	13	40	27	5	84	49.1	38.5	18	0.0075
	Aztreonam	51	29	36	24	8	84	40.5	27.4	37	0.0010
	Ceftazidime	75	5	36	58	0	58	49.1	46.2	5	1.0000
	Ciprofloxacin	60	6	50	57	9	50	50.9	35.9	15	0.0993
	Cefepime	67	9	40	26	3	87	40.5	48.7	12	0.8221
	Imipenem	61	7	48	51	0	65	62.9	30.8	7	0.0002
	Levofloxacin	51	10	55	51	9	56	50.0	34.2	19	0.3521
	Tobramycin	73	8	35	79	1	36	62.1	29.9	9	0.0132
	Piperacillin-tazobactam	69	8	39	46	7	63	48.7	37.6	15	0.0843
***Proteus mirabilis***	Amikacin	52	0	19	39	1	31	62.0	36.6	1	0.1048
	Ampicillin	23	0	48	32	0	39	62.0	38.0	0	0.0784
	Gentamicin	36	0	35	46	0	25	64.8	35.2	0	0.0263
	Cefotaxime	41	2	28	45	7	19	52.1	35.2	9	0.1451
	Cefepime	40	12	19	37	10	24	42.3	32.4	22	0.6368
	Cefoxitin	69	1	1	39	1	31	52.1	45.1	2	0.4978
	Imipenem	51	20	0	54	0	17	53.5	18.3	20	0.7624
	Levofloxacin	25	17	29	52	7	12	46.5	36.6	24	0.1667
	Tobramycin	33	18	20	51	2	18	52.1	22.5	20	0.0339

*S* Susceptible, *I* Intermediately resistant, *R* Resistant, *CA* Categorical agreement, *MD* Major discrepancies, *mD* Minor discrepancies

## Results

### Preclinical Experiment

**Fig. 2:**
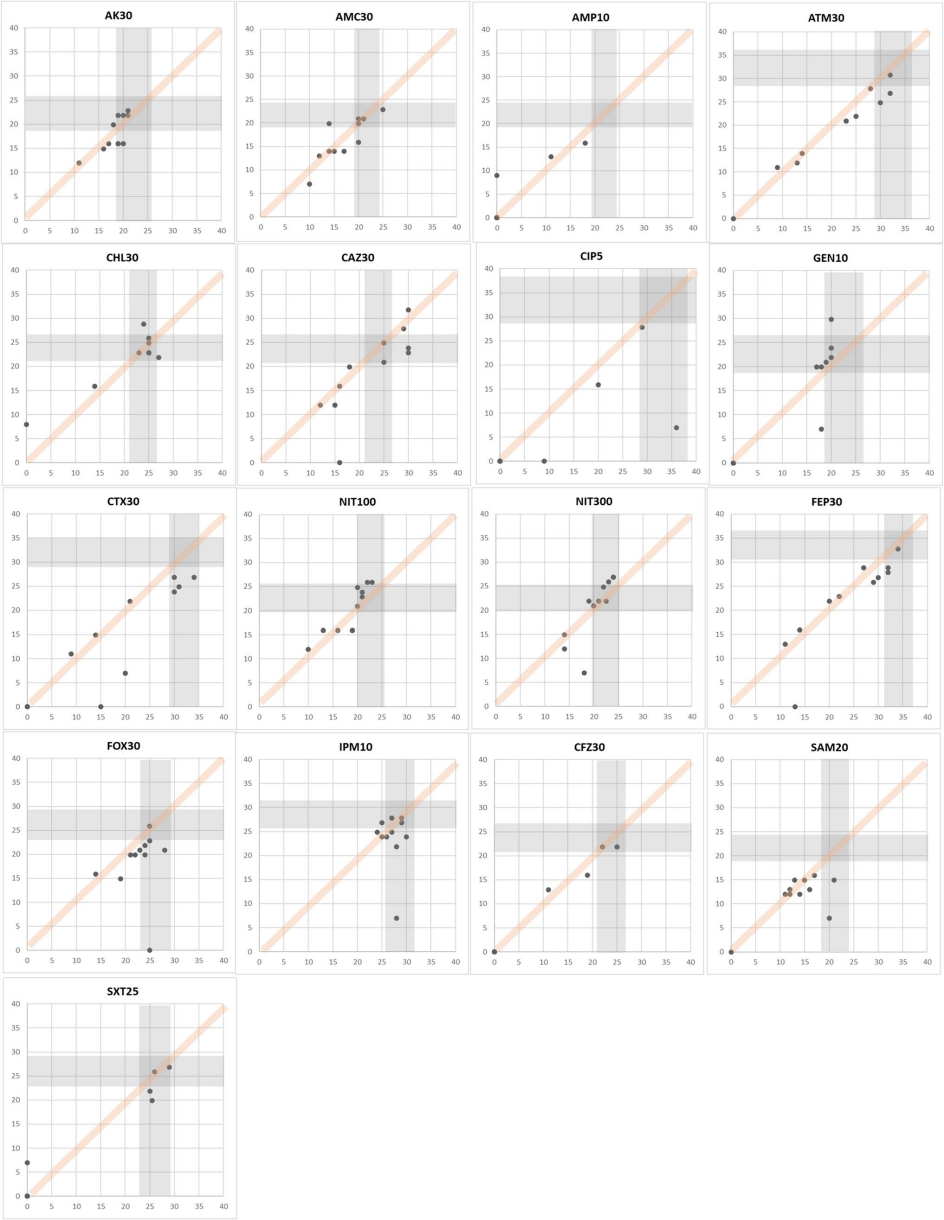
Scatter plots demonstrate the inhibition zone diameters for 17 antimicrobial susceptibilities of preclinically tested 11 *E. coli* strains. The X-axis shows the inhibition zone diameter tested by manual measurement, and the Y-axis shows the inhibition zone diameter from the RDD method. The areas for susceptible, intermediate, and resistant (S/I/R) categorization, as per CLSI breakpoint standards, are indicated in gray. Abbreviations: AK30, amikacin 30 μg; AMC30, amoxicillin-clavulanate 20/10 μg; AMP10, ampicillin 10 μg; ATM30, aztreonam 30 μg; CHL30, chloramphenicol 30 μg; CAZ30, ceftazidime 30 μg; CIP5, ciprofloxacin 5 μg; GEN10, gentamicin 10 μg; CTX30, cefotaxime 30 μg; NIT100, nitrofurantoin 100 μg; NIT300, nitrofurantoin 300 μg; FEP30, cefepime 30 μg; FOX30, cefoxitin 30 μg; IMP10, imipenem 10 μg; CFZ30, cefazoline 30 μg; SAM20, ampicillin-sulbactam 10/10 μg; SXT25, trimethoprim-sulfamethoxazole 1.25/23.75 μg

**Fig. 3:**
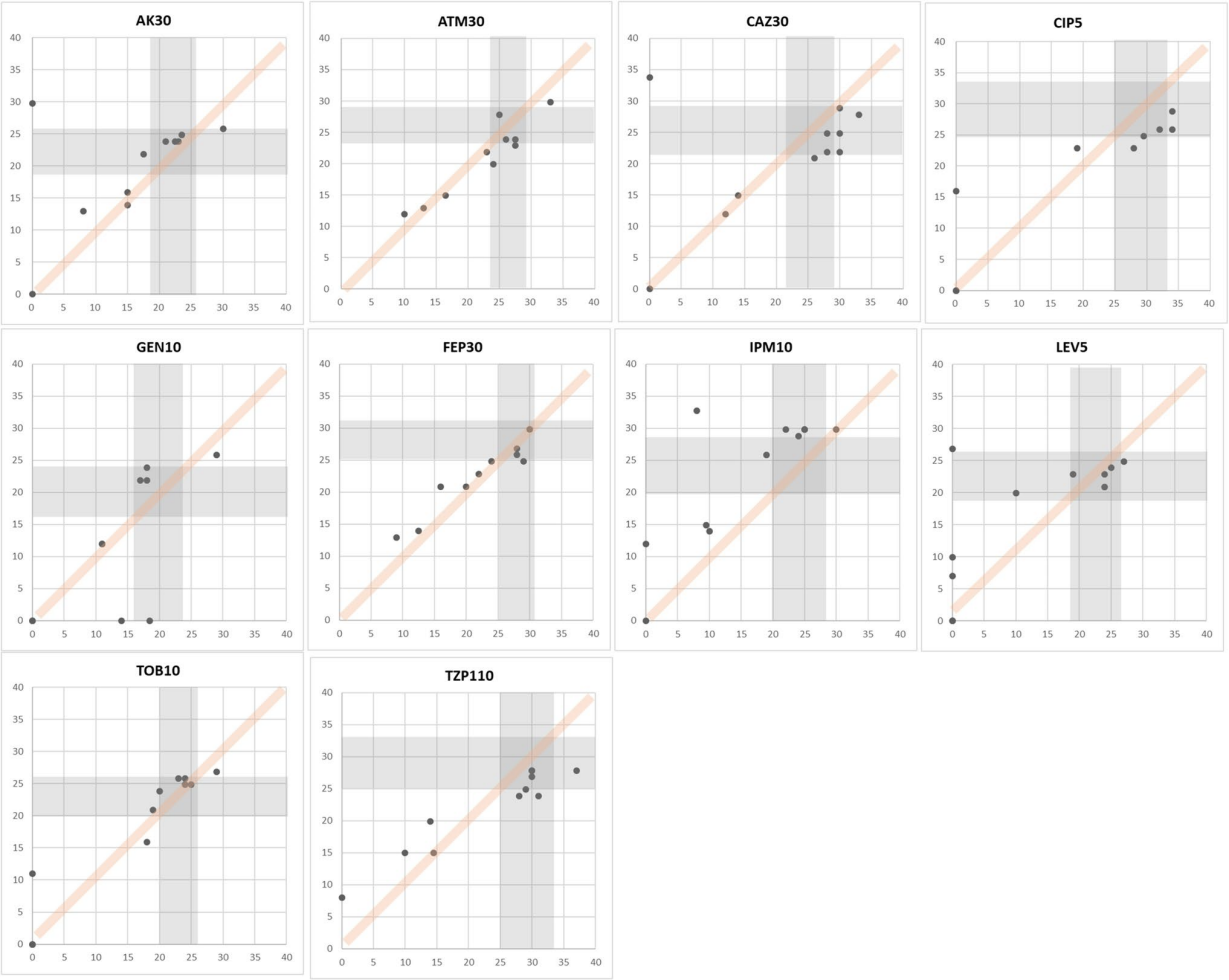
Scatter plots demonstrate the inhibition zone diameters for 10 antimicrobial susceptibilities of preclinically tested 11 *P. aeruginosa* strains. The X-axis shows the inhibition zone diameter tested by manual measurement, and the Y-axis shows the inhibition zone diameter from the RDD method. The areas for susceptible, intermediate, and resistant (S/I/R) categorization, as per CLSI breakpoint standards, are indicated in gray. Abbreviations: AK30, amikacin 30 μg; ATM30, aztreonam 30 μg; CAZ30, ceftazidime 30 μg; CIP5, ciprofloxacin 5 μg; GEN10, gentamicin 10 μg; FEP30, cefepime 30 μg; IMP10, imipenem 10 μg; LEV5, levofloxacin 5 μg; TOB10, tobramycin 10 μg; TZP110, piperacillin-tazobactam 100/10 μg

**Fig. 4:**
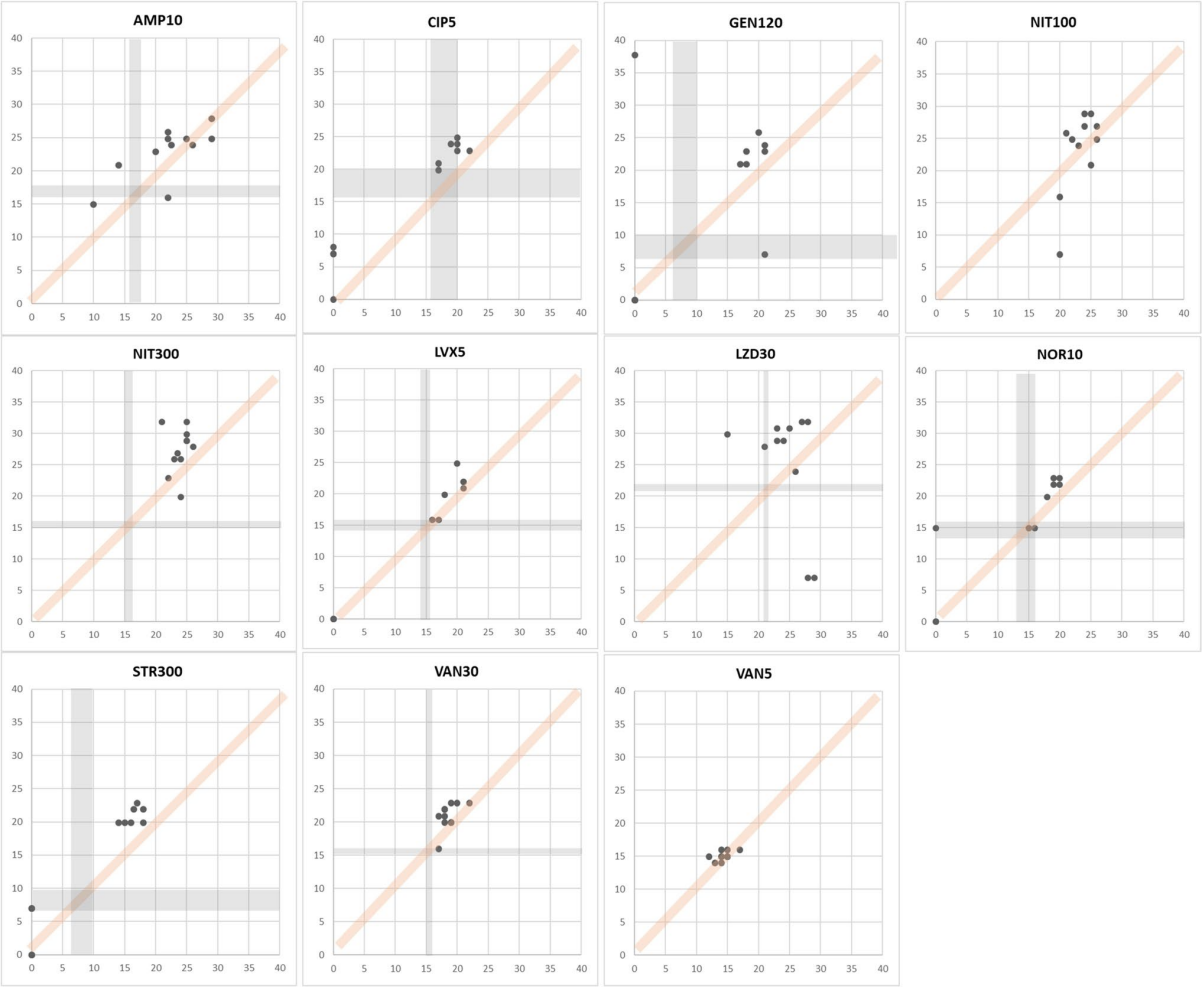
Scatter plots demonstrate the inhibition zone diameters for 11 antimicrobial susceptibilities of preclinically tested *E. faecalis* strains. The X-axis shows the inhibition zone diameter tested by manual measurement, and the Y-axis shows the inhibition zone diameter from the RDD method. The areas for susceptible, intermediate, and resistant (S/I/R) categorization, as per CLSI breakpoint standards, are indicated in gray. Abbreviations: AMP10, ampicillin 10 μg; CIP5, ciprofloxacin 5 μg; GEN120, gentamicin 120 μg; NIT100, nitrofurantoin 100 μg; NIT300, nitrofurantoin 300 μg; LEV5, levofloxacin 5 μg; LZD30, linezolid 30 μg; NOR10, norfloxacin 10 μg; STR300, streptomycin 300 μg; VAN30, vancomycin 30 μg; VAN5, vancomycin 5 μg

**Fig. 5:**
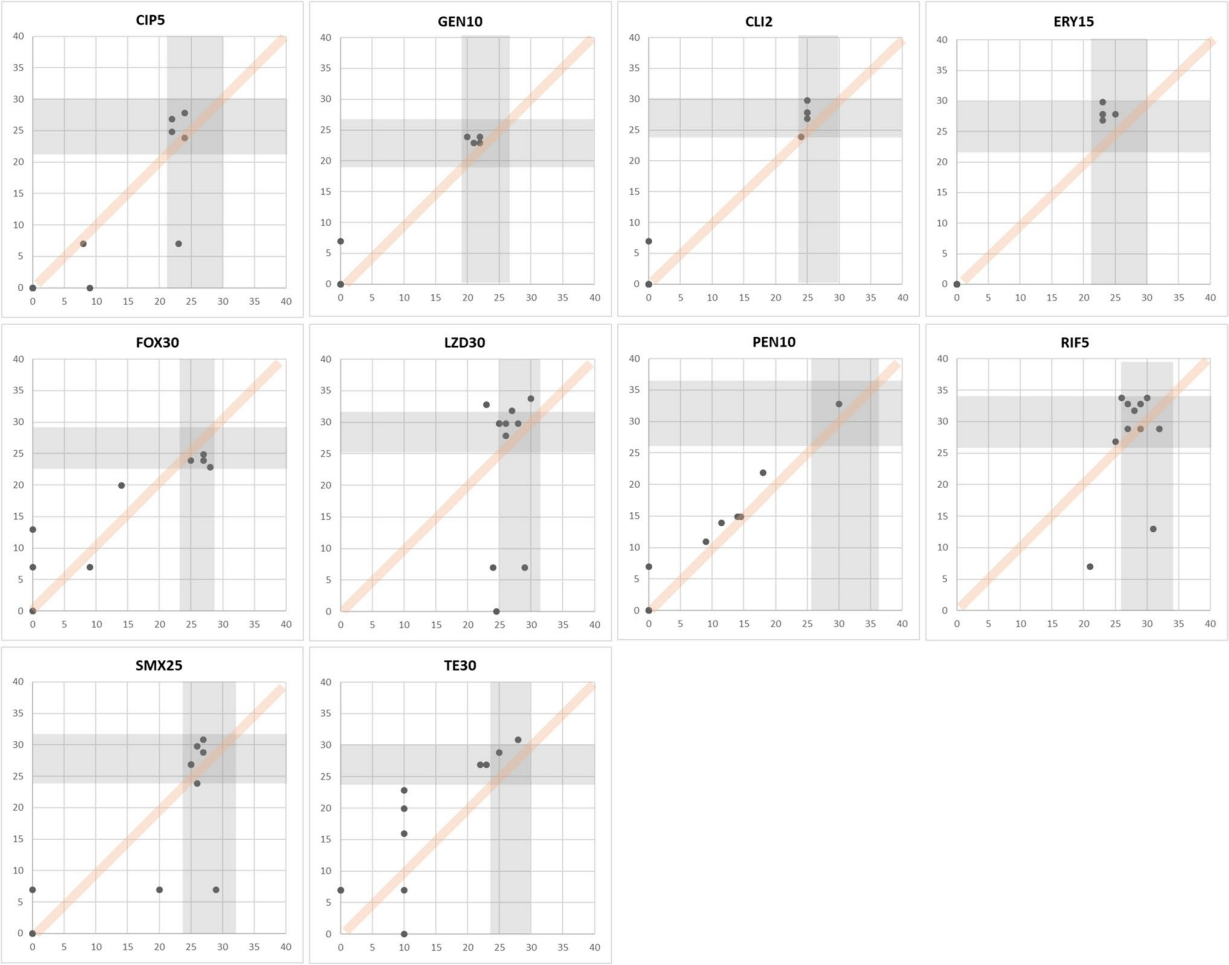
Scatter plots demonstrate the inhibition zone diameters for 10 antimicrobial susceptibilities of preclinically tested 11 *S. aureus* strains. The X-axis shows the inhibition zone diameter tested by manual measurement, and the Y-axis shows the inhibition zone diameter from the RDD method. The areas for susceptible, intermediate, and resistant (S/I/R) categorization, as per CLSI breakpoint standards, are indicated in gray. Abbreviations: CIP5, ciprofloxacin 5 μg; GEN10, gentamicin 10 μg; CLI2, clindamycin 2 μg; ERY15, erythromycin 15 μg; FOX30, cefoxitin 30 μg; LZD30, linezolid 30 μg; PEN10, penicillin 10 μg; RIF5, rifampin 5 μg; SMX25, trimethoprim-sulfamethoxazole 1.25/23.75 μg; TE30, tetracycline 30 μg

CAs play a crucial role in assessing antibiotic effectiveness by ensuring consistency and reliability in measuring antimicrobial inhibition zones. These agreements establish defined criteria for interpreting inhibition zones by considering the diameter of growth inhibition surrounding antimicrobial discs. A total of 187 data points were gained with eleven *E. coli* strains for 17 antimicrobials. Specifically, ampicillin and cefazolin demonstrated 100% CAs, while chloramphenicol, ciprofloxacin, nitrofurantoin (100 μg), cefepime, ampicillin-sulbactam, and trimethoprim-sulfamethoxazole showed 80–90%. Further, amikacin, amoxicillin-clavulanate, aztreonam, ceftazidime, gentamicin, cefotaxime, and nitrofurantoin (300 μg) demonstrated 60–70% CAs. However, the CAs for imipenem and cefoxitin showed significantly low CAs of 40–50%. MDs were absent. The data points acquired on antimicrobials are shown in Fig. 2. Like *E. coli*, 110 data points were gained with eleven *P. aeruginosa* strains for 10 antimicrobials. Observations suggest that cefepime and tobramycin showed 80–90% CAs, amikacin, aztreonam, gentamicin, levofloxacin, and piperacillin-tazobactam showed 60–70% CAs. Contrastingly, CAs for imipenem, ciprofloxacin, and ceftazidime were notably low as 40–50%. In this experiment with *P. aeruginosa* strains, nine MDs were observed. The data points acquired on antimicrobials are shown in Fig. 3. Further, 121 data points were gained with *E. faecalis* strains for 11 antimicrobials. Observations indicate that CAs were 100% for nitrofurantoin (300 μg) and 80–90% for ampicillin, gentamicin, levofloxacin, norfloxacin, streptomycin, and vancomycin. However, 50–60% of CAs were observed with ciprofloxacin and linezolid. There were seven MDs with these antimicrobials. The data points acquired on antimicrobials are shown in Fig. 4. Furthermore, 110 data points were gained with 11 *S. aureus* strains for 10 antimicrobials. Observations indicate that CAs were 100% for gentamicin, erythromycin, and penicillin, while ciprofloxacin, clindamycin, rifampin, cefoxitin, rifampin, and trimethoprim-sulfamethoxazole showed 80–90% CAs. Additionally, antimicrobials like linezolid and tetracycline showed approximately 70% CAs. There was only one MD observed with these antimicrobials. The data points acquired on antimicrobials are shown in Fig. 5. Collectively, these observations help clinicians make decisions regarding treatment options. Also, By employing this methodical approach, the precision of AST is improved, which in turn helps in the fight against AMR and facilitates the provision of efficient patient care.

### Clinical Experiment Outcome

In this study, a total of 2445 drug-organism combinations were observed with CA, MD, and mD in the clinical experiments with *S. aureus* and *E. faecalis*. The results indicated that 67.4% (1648/2445) CA, 29.6% (723/2445) of MDs, and 3.0% (74/2445) of mDs among manual measurements and RDD method. Similarly, 2736 drug-organism combinations were observed with CA, MD, and mD in the clinical experiments with *K. pneumoniae*, *P. aeruginosa,* and *P. mirabilis*. Here, observations showed 51.1% (1398/2736) CA, 34.8% (952/2736) of MDs, and 15.6% (428/2736) of mDs among manual measurements and RDD method. All the relevant observations are described in the Table 1 and Supplementary Tables. In *S. aureus*, the CAs were more than 70% for ciprofloxacin, gentamicin, clindamycin, linezolid, rifampin, trimethoprim-sulfamethoxazole, and tetracycline. Categorizations of ciprofloxacin, gentamicin and erythromycin are significantly different between manual measurements and RDD methods. Further, observations indicated high sensitivity and high PPV, suggesting that the LS-AST method is highly effective in identifying *S. aureus*. However, specificity and NPV were observed to be low for most antibiotics, suggesting a high rate of false resistance. Interestingly, antimicrobials such as gentamicin exhibited a high PLR and a low NLR, indicating their impressive effectiveness in AST. In *E. faecalis*, the CAs were around 50–60% for most antimicrobials. In addition, high PPV suggests the LS-AST methods effectiveness in confirming susceptible organisms. However, most antimicrobials have low specificity and low NPV, which indicate the considerable possibility of false resistance. In *K. pneumoniae*, the CA for gentamicin was observed to be highest at 63.2% and lowest observed with cefoxitin (36.8%). Other antimicrobials showed 40–50% of CAs. Observations on diagnostic tests indicated that the LS-AST method has low sensitivity and moderate to high specificity in most antimicrobials, suggesting LS-AST technique is efficient in identifying non-susceptible strains in most antimicrobials. Furthermore, *P. aeruginosa* demonstrated 62% CAs for imipenem and tobramycin, while both antimicrobials are significantly different for the susceptibility categorization between manual measurements and RDD methods. In *P. mirabilis*, the CAs for amikacin, ampicillin, and gentamicin were observed to be 60%, while other antimicrobials demonstrated 40–50% CAs. These observations on CA, MD, and mD in clinical setting suggest the efficiency of LS-AST methods. Moreover, most of the diagnostic test parameters in *P. aeruginosa* and *P. mirabilis* demonstrated promising values for carious antimicrobials. However, additional data are required to fully evaluate the effectiveness of LS-AST based on the specificities and other metrics.

## Discussion

Bacterial strains are progressively acquiring AMR, which has become a global concern. Excessive and inappropriate use of antimicrobials accelerated this development of AMR, rendering once-effective treatments ineffective. Over the years, multiple strategies were developed to address the AMR crisis. Among these, rapid AST methods are prominent, and they assist in providing customized antibiotic treatment regimens to patients [21]. This regulates the irrational use of antibiotics. However, the turnaround time of the conventional AST acts as a hurdle and forces physicians to take calls on the use of antimicrobials depending on their clinical judgment. Multiple AST methods that rely on high-resolution imaging and time-lapse surveys have been developed, but most are considered expensive for routine operations. In this study, an established LSF technology was applied for quick and effective results. As expected, preclinical and clinical studies demonstrated the efficacy of the LS-AST method in determining antimicrobial susceptibility in different bacterial strains. Specifically, the majority of the antimicrobials used in the preclinical studies demonstrated > 80% CAs (Fig. 5). These observations were primarily reflected in the clinical studies where CAs were as high as 78% (Table 1). These observations strongly suggest the potential application of LS-AST device in rapid AST.

The level of accuracy in the study demonstrates that the LS-AST method is robust against phenotype plasticity displayed by various clinical isolates such as *E. coli*, *S. aureus*, *E. faecalis*, *K. pneumoniae*, and *P. aeruginosa*. In addition, the LS-AST device determined antimicrobial susceptibility in the shortest possible time of 4 h, which is among the quickest AST methods. Undoubtedly, a reduced turnaround time benefits physician in prescribing the appropriate antimicrobial at the right concentration. These prospective benefits were realized as a result of the instrument's design. Specifically, speckles are known to display sensitivity to phase and amplitude variations introduced by bacteria [22]. Moreover, in the presence of a colony, scattering occurs when a laser with a parallel wavefront passes through a colony. This scattered light and the wavefront interfere with the laser with a parallel wavefront, producing speckle patterns. These patterns respond to changes in optical path length in microbial samples, which is better than the camera-based AST, such as single-cell technologies [23]. Together, the design of the LS-AST helps maintain the necessary optical characteristics without compromising its performance. Interestingly, the method detects bacterial growth, which is invisible to the eye by spatial correlation coefficient, which is highly sensitive to subtle changes in bacterial growth patterns. This is considered to be a high-throughput screening method. Collectively, the LS-AST device design shows greater efficacy in the identification of antimicrobial susceptibility.

The present antimicrobial susceptibility measured with LS-AST showed notable preclinical and clinical experiments observations. CA in antimicrobial susceptibility testing is crucial for the accurate interpretation of observations. Consistency between the results of the different tests is essential, given that susceptibility categorization can significantly influence the physician's treatment decision and, consequently, the course of action taken with patients [24]. In preclinical experiments, ampicillin, ampicillin-sulbactam, and aztreonam demonstrated 100%, 81 and 80% CAs, respectively, against *E. coli*. Similarly, cephalosporin and cefazolin showed a high CA rate of 100%, while ceftazidime and cefepime showed 80% CAs. These high CA in antimicrobial susceptibility testing strongly indicate the efficacy of LS-AST method in preclinical experiments. However, clinical experiments with multiple organisms indicated lower CAs than the preclinical experiments. The discrepancy in outcomes between preclinical and clinical experiments is likely because preclinical experiments were based on resistant strains and reference strains, while clinical experiments were based on clinical strains, including susceptible strains. The evaluation of CAs are crucial in assessing antibiotic effectiveness by ensuring consistency and reliability in measuring antimicrobial inhibition zones. Observations on MD and mD reveal significant discrepancies in the outcomes obtained from different testing methodologies. Identifying MD and mD is essential to ensuring consistency and accuracy while evaluating the effectiveness of antibiotics. Moreover, it allows researchers and doctors to identify potential reasons for error and establish standardized testing processes. Also, clinical studies offer essential data regarding the device's efficacy compared to conventional methods, guiding its adoption in routine practice.

The preclinical and clinical studies showed the efficiency of LS-AST approach. In addition, design of the equipment is adjusted to captures the speckle pattern when the laser penetrates the sample. The equipment setup in transmission mode prevents the laser from penetrating the area beneath the antibiotic disk. Thus, it is possible to precisely establish the form and position of the disk, allowing accurately quantify the diameter of the inhibitory zone. This undoubtedly represents an improvement above the previous similar methods [10]. Besides, the method is not influenced by the mechanical movements as image shift does not interfere with spatial correlation [17]. Undoubtedly, it is quick and potentially accurate but may be prone to a few sources of error while measuring the inhibitory zone. First, there may be an unstable response to antimicrobials (colony death after measurement). Contrastingly, there may be initial susceptibility to antimicrobials, but over time, growth could occur. Secondly, the assay may be distorted due to unpredictable changes in the culture medium, such as drying of the culture medium. This kind of unpredictable variations potentially influence the diffraction pattern, leading to errors in the measurement of the inhibition zone. Moreover, uneven distribution and smaller bacterial colonies with slow growth rates might affect the inhibition zone measurement by the equipment. Thus, further study using the LS-AST is required in order to address the challenges identified in the present study. In addition, evaluating the Minimum Inhibitory Concentration (MIC) can significantly enhance the findings obtained from the LS-AST. However, due to their fundamental differences in evaluation, MIC cannot be performed with disk diffusion methods. Therefore, an independent investigation of MIC could help validate the LS-AST observations.

## Conclusion

AST holds profound significance in determining the most effective treatment for bacterial infections. However, physicians face the greatest challenge in prescribing the appropriate antimicrobials due to the turnaround time for ASTs. In this study, LSF technology was utilized to develop a new device to determine antimicrobial susceptibility. The approach displayed significant accuracy across diverse bacterial strains and antimicrobial drugs, suggesting its potential applicability to various bacterial strains.The proposed method and design exhibit greater application potential than previously developed comparable methods. The current study is marked by a unique equipment setup and analysis approach. This distinction contributes to analyzing the inhibition zones precisely. In addition, study significantly contribute to the ongoing effort to develop faster AST approaches that are essential for addressing AMR in clinical settings.

### Supplementary Information

Below is the link to the electronic supplementary material.

Supplementary file1 (DOCX 57 kb)

## Data Availability

The datasets generated during and/or analysed during the study are available from the corresponding author upon reasonable request.

## Abbreviations

AMR: Antimicrobial resistance
AST: Antimicrobial susceptibility testing
ATCC: American Type Culture Collection
CA: Category agreements
CCARM: Culture Collection of Antimicrobial Resistant Microbes
DD: Disk diffusion
EUCAST: European Committee on antimicrobial susceptibility testing
LS-AST: Laser scattering-based antimicrobial susceptibility test
LSF: Laser speckle formation
MD: Major discrepancy
mD: Minor discrepancies
MIC: Minimum inhibitory concentration
MHA: Mueller–Hinton agar
RDD: Rapid disk diffusion

## References

[1] van Belkum A, Bachmann TT, Lüdke G, Lisby JG, Kahlmeter G, Mohess A et al (2019) Developmental roadmap for antimicrobial susceptibility testing systems. Nat Rev Microbiol 17(1):51–62. 10.1038/s41579-018-0098-9 30333569 10.1038/s41579-018-0098-9 PMC7138758

[2] Reller LB, Weinstein M, Jorgensen JH, Ferraro MJ (2009) Antimicrobial susceptibility testing: a review of general principles and contemporary practices. Clin Infect Dis 49(11):1749–1755 19857164 10.1086/647952

[3] Brown C, Tseng D, Larkin PMK, Realegeno SE, Di Carlo D, Garner OB et al (2020) An automated and cost-effective system for early antimicrobial susceptibility testing. In: Conference on lasers and electro-optics, Optica Publishing Group, Washington, DC, p AM3I.6

[4] Ledeboer NA, Hodinka RL (2011) Molecular detection of resistance determinants. J Clin Microbiol 49(9):S20–S24

[5] Kaprou GD, Bergspica I, Alexa EA, Alvarez-Ordonez A, Prieto M (2021) Rapid methods for antimicrobial resistance diagnostics. Antibiotics (Basel). 10.3390/antibiotics10020209 33672677 10.3390/antibiotics10020209 PMC7924329

[6] Reynoso EC, Laschi S, Palchetti I, Torres E (2021) Advances in antimicrobial resistance monitoring using sensors and biosensors: a review. Chemosensors 9(8):232 10.3390/chemosensors9080232

[7] Shams S, Lima C, Xu Y, Ahmed S, Goodacre R, Muhamadali H (2023) Optical photothermal infrared spectroscopy: a novel solution for rapid identification of antimicrobial resistance at the single-cell level via deuterium isotope labeling. Front Microbiol 14:1077106. 10.3389/fmicb.2023.1077106 36819022 10.3389/fmicb.2023.1077106 PMC9929359

[8] Vasala A, Hytönen VP, Laitinen OHJF, Microbiology I (2020) Modern tools for rapid diagnostics of antimicrobial resistance. Front Cell Infect Biol 10:308 10.3389/fcimb.2020.00308 PMC7373752 32760676

[9] Jonasson E, Matuschek E, Kahlmeter G (2020) The EUCAST rapid disc diffusion method for antimicrobial susceptibility testing directly from positive blood culture bottles. J Antimicrob Chemother 75(4):968–978. 10.1093/jac/dkz548 32016342 10.1093/jac/dkz548 PMC7069491

[10] Balmages I, Reinis A, Kistkins S, Bliznuks D, Plorina EV, Lihachev A et al (2023) Laser speckle imaging for visualization of hidden effects for early detection of antibacterial susceptibility in disc diffusion tests. Front Microbiol 14:1221134. 10.3389/fmicb.2023.1221134 37455709 10.3389/fmicb.2023.1221134 PMC10340531

[11] Khan B, Fatima H, Qureshi A, Kumar S, Hanan A, Hussain J et al (2023) Drawbacks of artificial intelligence and their potential solutions in the healthcare sector. Biomed Mater Devices. 10.1007/s44174-023-00063-2 36785697 10.1007/s44174-023-00063-2 PMC9908503

[12] Gianfrancesco MA, Tamang S, Yazdany J, Schmajuk G (2018) Potential biases in machine learning algorithms using electronic health record data. JAMA Intern Med 178(11):1544–1547. 10.1001/jamainternmed.2018.3763 30128552 10.1001/jamainternmed.2018.3763 PMC6347576

[13] Tsamados A, Aggarwal N, Cowls J, Morley J, Roberts H, Taddeo M et al (2022) The ethics of algorithms: key problems and solutions. AI Soc. 10.1007/s00146-021-01154-8 10.1007/s00146-021-01154-8

[14] Curtoni A, Ghibaudo D, Veglio C, Imperatore L, Bianco G, Castiglione A et al (2023) Light scattering technology and MALDI-TOF MS in the microbiological fast-track of bloodstream infections: potential impact on antimicrobial treatment choices in a real-life setting. J Med Microbiol. 10.1099/jmm.0.001638 36748537 10.1099/jmm.0.001638

[15] Xu Z, Liu J, Hong DH, Nguyen VQ, Kim MR, Mohammed SI et al (2010) Back-directional gated spectroscopic imaging for diffuse light suppression in high anisotropic media and its preclinical applications for microvascular imaging. IEEE J Sel Top Quantum Electron 16(4):815–823. 10.1109/JSTQE.2009.2037160 10.1109/JSTQE.2009.2037160

[16] Zhang EZ, Povazay B, Laufer J, Alex A, Hofer B, Pedley B et al (2011) Multimodal photoacoustic and optical coherence tomography scanner using an all optical detection scheme for 3D morphological skin imaging. Biomed Opt Express 2(8):2202–2215. 10.1364/BOE.2.002202 21833358 10.1364/BOE.2.002202 PMC3149519

[17] Han S, No H, Baek Y, Park H, Lee K, Yang S et al (2019) Rapid antimicrobial susceptibility test using spatiotemporal analysis of laser speckle dynamics of bacterial colonies

[18] Han S, Kim H, Park J, Lee S, Lee K, Kim J-K et al (2018) Real-time monitoring of bacterial growth and fast antimicrobial susceptibility tests exploiting multiple light scattering 481184

[19] Melvin P, Weinstein M (2018) Performance standards for antimicrobial disk susceptibility tests. Clinical & Laboratory Standards Institute

[20] Tamma PD, Harris PNA, Mathers AJ, Wenzler E, Humphries RM (2023) Breaking down the breakpoints: rationale for the 2022 clinical and laboratory standards institute revised piperacillin-tazobactam breakpoints against enterobacterales. Clin Infect Dis 77(11):1585–1590. 10.1093/cid/ciac688 36001445 10.1093/cid/ciac688

[21] do Jorgensen JJD (2009) MJ Ferraro, Antimicrobial susceptibility testing: a review of general principles and contemporary practices. Clin Infect Dis 49:1749–1755 19857164 10.1086/647952

[22] Suchwalko A, Buzalewicz I, Podbielska H (2014) Bacteria identification in an optical system with optimized diffraction pattern registration condition supported by enhanced statistical analysis. Opt Express 22(21):26312–26327. 10.1364/OE.22.026312 25401664 10.1364/OE.22.026312

[23] Le Page S, Raoult D, Rolain JM (2015) Real-time video imaging as a new and rapid tool for antibiotic susceptibility testing by the disc diffusion method: a paradigm for evaluating resistance to imipenem and identifying extended-spectrum beta-lactamases. Int J Antimicrob Agents 45(1):61–65. 10.1016/j.ijantimicag.2014.08.014 25455851 10.1016/j.ijantimicag.2014.08.014

[24] Temmerman R, Goethals K, Garmyn A, Vanantwerpen G, Vanrobaeys M, Haesebrouck F et al (2020) Agreement of quantitative and qualitative antimicrobial susceptibility testing methodologies: the case of enrofloxacin and avian pathogenic *Escherichia coli*. Front Microbiol 11:570975. 10.3389/fmicb.2020.570975 33042075 10.3389/fmicb.2020.570975 PMC7525152

