# Supplementary material for: Development of Rapid Disk Diffusion Device Using Laser Speckle Formation Technology for Rapid Antimicrobial Susceptibility Testing
Source: Curr Microbiol. 2024 Jul 14;81(9):269. doi: 10.1007/s00284-024-03798-3 (PMC11247048; doi:10.1007/s00284-024-03798-3)
Supplement: Supplementary file 1 — Supplementary file1 (DOCX 57 kb) [file 284_2024_3798_MOESM1_ESM.docx]

**Development of Rapid Disk Diffusion Device using Laser Speckle Formation Technology for Rapid Antimicrobial Susceptibility testing**

Jaehyeon Lee^1,2^, Jun Han Lee^2^, Kyoungman Cho^3*^, Jeong Su Park^4*^

^1^ Department of Laboratory Medicine, Jeonbuk National University Medical School and Hospital, Jeonju, Republic of Korea

^2^ Research Institute of Clinical Medicine of Jeonbuk National University-Biomedical Research Institute of Jeonbuk National University Hospital, Jeonju, Republic of Korea

^3^ The Wave Talk., Inc., Jinri hall, 193, Munji-ro, Yueseong-gu, Daejeon 34051, Republic of Korea

^4^ Department of Laboratory Medicine, Seoul National University Bundang Hospital, Seoul National University College of Medicine, Seongnam, Republic of Korea

***Correspondence: Jeong Su Park**, Department of Laboratory Medicine, Seoul National University Bundang Hospital, Seoul National University College of Medicine, Seongnam, Republic of Korea. Email: mdmicrobe@gmail.com. **Kyoungman Cho**, The Wave Talk, Inc., Jinri hall, 193, Munji-ro, Yueseong-gu, Daejeon 34051, Repulic of Korea. *Email:**kyoungman-cho@thewavetalk.com*.

**SUPPLEMENTARY TABLES**

**Table 1.** Percentage of sensitivity, specificity, PPV, and NPV of the LS-AST method in comparison to manual method in *Staphylococcus aureus* against different antibiotics.

|  | Sensitivity | | Specificity | | PPV | | NPV | |
| --- | --- | --- | --- | --- | --- | --- | --- | --- |
|  | % | 95% CI | % | 95% CI | % | 95% CI | % | 95% CI |
| Ciprofloxacin | 86.2 | 79.2 – 91.1 | 36.4 | 19.7 – 57.1 | 88.9 | 82.2 – 93.3 | 30.8 | 16.5 – 50.0 |
| Gentamicin | 82.0 | 74.2 –87.8 | 66.7 | 48.8 – 80.8 | 90.9 | 84.1 – 95.0 | 47.6 | 33.4 – 62.3 |
| Clindamycin | 81.5 | 74.4 –87.0 | 16.7 | 3.0 – 56.4 | 96.0 | 90.9 – 98.3 | 3.6 | 0.6 – 17.7 |
| Erythromycin | 80.5 | 72.3 –86.8 | 43.6 | 29.3 – 59.0 | 80.5 | 72.3 – 86.8 | 43.6 | 29.3 – 59.0 |
| Cefoxitin | 48.0 | 38.3 –57.7 | 55.6 | 42.4 – 68.0 | 66.2 | 54.6 – 76.1 | 37.0 | 27.3 – 47.9 |
| Linezolid | 76.3 | 69.0 –82.4 | NC | NC | 100.0 | 96.8 – 100.0 | 0.0 | 0.0 – 9.6 |
| Rifampin | 78.3 | 71.1 –84.1 | NC | NC | 100.0 | 96.9 – 100.0 | 0.0 | 0.0 – 10.4 |
| Trimethoprim-sulfamethoxazole | 76.3 | 69.0 –82.4 | NC | NC | 100.0 | 96.8 – 100.0 | 0.0 | 0.0 – 9.6 |
| Tetracycline | 79.7 | 72.5 –85.4 | 33.3 | 6.2 – 79.2 | 98.3 | 94.1– 99.5 | 3.2 | 0.6 – 16.2 |

PPV: Positive predictive value; NPV: Negative predictive value; CI: Confidence interval; NC: Not calculable.

**Table 2.** Accuracy, PLR, NLR, and DOR of the LS-AST method in comparison to manual method in *Staphylococcus aureus* against different antibiotics.

|  | Accuracy | | PLR | | NLR | | DOR | |
| --- | --- | --- | --- | --- | --- | --- | --- | --- |
|  | % | 95% CI |  | 95% CI |  | 95% CI |  | 95% CI |
| Ciprofloxacin | 79.0 | 71.8 – 84.7 | 1.35 | 0.47 – 3.92 | 0.38 | 0.13 – 1.10 | 3.56 | 0.79 – 15.96 |
| Gentamicin | 79.0 | 71.8 – 84.7 | 2.46 | 1.07 – 5.64 | 0.27 | 0.12 – 0.62 | 9.09 | 2.81 – 29.41 |
| Clindamycin | 79.0 | 71.8 – 84.7 | 0.98 | 0.09 – 10.35 | 1.11 | 0.10 – 11.74 | 0.88 | 0.03 – 24.78 |
| Erythromycin | 71.1 | 63.4 – 77.7 | 1.43 | 0.61 – 3.33 | 0.45 | 0.19 – 1.04 | 3.20 | 0.97 – 10.57 |
| Cefoxitin | 50.7 | 42.8 – 58.5 | 1.08 | 0.49 – 2.39 | 0.94 | 0.42 – 2.07 | 1.15 | 0.37 – 3.54 |
| Linezolid | 76.3 | 69.0 – 82.4 | 0.76 | 0.60 – 0.97 | NC | NC | NC | NC |
| Rifampin | 78.3 | 71.1 – 84.1 | 0.78 | 0.62 – 1.00 | NC | NC | NC | NC |
| Trimethoprim-sulfamethoxazole | 76.3 | 69.0 – 82.4 | 0.76 | 0.60 – 0.97 | NC | NC | NC | NC |
| Tetracycline | 78.8 | 71.6 – 84.6 | 1.20 | 0.10 – 14.64 | 0.61 | 0.05 – 7.45 | 1.97 | 0.06 – 67.97 |

PLR: Positive likelihood ratio; NLR: Negative likelihood ratio: DOR: Diagnostic odds ratio; CI: Confidence interval; NC: Not calculable.

**Table 3.** Percentage of sensitivity, specificity, PPV, and NPV of the LS-AST method in comparison to manual method in *Enterococcus faecalis* against different antibiotics.

|  | Sensitivity | | Specificity | | PPV | | NPV | |
| --- | --- | --- | --- | --- | --- | --- | --- | --- |
|  | % | 95% CI | % | 95% CI | % | 95% CI | % | 95% CI |
| Ampicillin | 69.2 | 61.2 – 76.2 | 54.5 | 28.0 – 78.7 | 95.2 | 89.2 – 97.9 | 12 | 5.6 – 23.8 |
| Gentamicin (120 µg) | 59.5 | 50.4 – 68.0 | 55.3 | 39.7 – 69.9 | 80.2 | 70.6 – 87.3 | 30.9 | 21.2 – 42.6 |
| Nitrofurantoin (300 µg) | 59.7 | 51.8 – 67.2 | NC | NC | 100.0 | 96.0 – 100.0 | 0.0 | 0.0 – 5.8 |
| Linezolid | 56.9 | 48.8 – 64.7 | 20.0 | 5.7 – 51.0 | 91.1 | 83.4 – 95.4 | 3.1 | 0.9 – 10.7 |
| Streptomycin (300 µg) | 60.8 | 52.8 – 68.3 | 100.0 | 61..0 – 100.0 | 100.0 | 95.9 – 100.0 | 9.4 | 4.4 – 19.0 |
| Teicoplanin | 60.8 | 52.9 – 68.2 | 0.0 | 0.0 – 79.3 | 98.9 | 94.3 – 99.8 | 0.0 | 0.0 – 6.0 |
| Vancomycin | 61.4 | 53.5 – 68.8 | 0.0 | 0.0 – 79.3 | 98.9 | 94.3 – 99.8 | 0.0 | 0.0 – 6.1 |

PPV: Positive predictive value; NPV: Negative predictive value; CI: Confidence interval; NC: Not calculable.

**Table 4.** Accuracy, PLR, NLR, and DOR of the LS-AST method in comparison to manual method in *Enterococcus faecalis* against different antibiotics.

|  | Accuracy | | PLR | | NLR | | DOR | |
| --- | --- | --- | --- | --- | --- | --- | --- | --- |
|  | % | 95% CI |  | 95% CI |  | 95% CI |  | 95% CI |
| Ampicillin | 68.2 | 60.5 – 75.0 | 1.52 | 0.79 – 2.94 | 0.56 | 0.31 – 1.02 | 2.70 | 0.78 – 9.32 |
| Gentamicin (120 µg) | 58.4 | 50.5 – 65.9 | 1.33 | 0.91 – 1.95 | 0.73 | 0.51 – 1.05 | 1.81 | 0.87 – 3.80 |
| Nitrofurantoin (300 µg) | 59.7 | 51.8 – 67.2 | NC | NC | NC | NC | NC | NC |
| Linezolid | 54.5 | 46.7 – 62.2 | 0.71 | 0.51 – 1.00 | 2.15 | 0.61 – 7.54 | 0.33 | 0.07 – 1.61 |
| Streptomycin (300 µg) | 62.3 | 54.5 – 69.6 | NC | NC | 0.39 | 0.32 – 0.48 | NC | NC |
| Teicoplanin | 60.4 | 52.5 – 67.8 | 0.61 | 0.54 – 0.69 | NC | NC | NC | NC |
| Vancomycin | 61.0 | 53.2 – 68.4 | 0.61 | 0.54 – 0.70 | NC | NC | NC | NC |

PLR: Positive likelihood ratio; NLR: Negative likelihood ratio: DOR: Diagnostic odds ratio; CI: Confidence interval; NC: Not calculable.

**Table 5.** Percentage of sensitivity, specificity, PPV, and NPV of the LS-AST method in comparison to manual method in *Klebsiella pneumoniae* against different antibiotics.

|  | Sensitivity | | Specificity | | PPV | | NPV | |
| --- | --- | --- | --- | --- | --- | --- | --- | --- |
|  | % | [95% CI] | % | [95% CI] | % | [95% CI] | % | [95% CI] |
| Ceftazidime | 27.6 | 16.7 – 40.9 | 79.7 | 67.2 – 89.0 | 57.1 | 40.9 – 72.0 | 52.8 | 47.7 – 57.9 |
| Ciprofloxacin | 19.4 | 8.2 – 36.0 | 97.5 | 91.4 – 99.7 | 77.8 | 43.3 – 94.1 | 73.2 | 69.8 – 76.3 |
| Gentamicin | 68.4 | 56.9 – 78.4 | 55.3 | 38.3 – 71.4 | 76.1 | 68.4 – 82.3 | 45.7 | 35.3 – 56.4 |
| Cefotaxime | 21.7 | 11.0 – 36.4 | 85.9 | 75.6 – 93.0 | 50 | 31.1 – 68.9 | 62.9 | 58.6 – 67.0 |
| Cefepime | 33.3 | 20.4 – 48.4 | 85.5 | 75.0 – 92.8 | 61.5 | 44.3 – 76.3 | 64.8 | 59.6 – 69.7 |
| Cefoxitin | 22.6 | 14.2 – 33.1 | 84.9 | 68.1 – 94.9 | 79.2 | 60.7 – 90.3 | 30.1 | 26.4 – 34.1 |
| Imipenem | 41.8 | 32.0 – 52.2 | 63.2 | 38.4 – 83.7 | 85.4 | 75.7 – 91.7 | 17.4 | 12.6 – 23.6 |
| Cefazolin | 21.9 | 9.3 – 40.0 | 57.7 | 46.5 – 68.3 | 16.3 | 8.8 – 28.1 | 66.2 | 60.2 – 71.7 |
| Ampicillin-sulbactam | 17.7 | 6.8 – 34.5 | 72.3 | 61.4 – 81.6 | 20.7 | 10.4 – 36.9 | 68.2 | 63.6 – 72.5 |

PPV: Positive predictive value; NPV: Negative predictive value; CI: Confidence interval.

**Table 6.** Accuracy, PLR, NLR, and DOR of the LS-AST method in comparison to manual method in *Klebsiella pneumoniae* against different antibiotics.

|  | Accuracy | | PLR | | NLR | | DOR | |
| --- | --- | --- | --- | --- | --- | --- | --- | --- |
|  | % | [95% CI] |  | [95% CI] |  | [95% CI] |  | [95% CI] |
| Ceftazidime | 53.9 | 44.4 – 63.1 | 1.36 | 0.70 – 2.61 | 0.91 | 0.74 – 1.12 | 1.47 | 0.63 – 3.51 |
| Ciprofloxacin | 73.5 | 64.6 – 81.2 | 7.88 | 1.72 – 36.07 | 0.83 | 0.70 – 0.97 | 9.53 | 1.87 – 48.57 |
| Gentamicin | 64.1 | 54.7 – 72.8 | 1.53 | 1.04 – 2.24 | 0.57 | 0.37 – 0.88 | 2.67 | 1.20 – 5.91 |
| Cefotaxime | 60.7 | 51.2 – 69.6 | 1.54 | 0.70 – 3.42 | 0.91 | 0.76 – 1.09 | 1.69 | 0.64 – 4.46 |
| Cefepime | 64.1 | 54.7 – 72.8 | 2.3 | 1.14 – 4.63 | 0.78 | 0.62 – 0.97 | 2.95 | 1.20 – 7.25 |
| Cefoxitin | 40.2 | 31.2 – 49.6 | 1.49 | 0.61 – 3.67 | 0.91 | 0.76 – 1.10 | 1.64 | 0.56 – 4.82 |
| Imipenem | 45.3 | 36.1 – 54.8 | 1.14 | 0.60 – 2.14 | 0.92 | 0.63 – 1.35 | 1.23 | 0.45 – 3.4 |
| Cefazolin | 47.9 | 38.5 – 57.3 | 0.52 | 0.26 – 1.04 | 1.36 | 1.05 – 1.75 | 0.38 | 0.15 – 0.98 |
| Ampicillin-sulbactam | 56.4 | 46.9 – 65.6 | 0.64 | 0.28 – 1.42 | 1.14 | 0.93 – 1.40 | 0.56 | 0.20 – 1.53 |

PLR: Positive likelihood ratio; NLR: Negative likelihood ratio: DOR: Diagnostic odds ratio; CI: Confidence interval.

**Table 7.** Percentage of sensitivity, specificity, PPV, and NPV of the LS-AST method in comparison to manual method in *Pseudomonas aeruginosa* against different antibiotics.

|  | Sensitivity | | Specificity | | PPV | | NPV | |
| --- | --- | --- | --- | --- | --- | --- | --- | --- |
|  | % | [95% CI] | % | [95% CI] | % | [95% CI] | % | [95% CI] |
| Amikacin | 77.8 | 57.7 – 91.4 | 52.8 | 41.9 – 63.5 | 33.3 | 27.1 – 40.3 | 88.7 | 79.0 – 94.2 |
| Aztreonam | 75.0 | 53.3 – 90.2 | 64.1 | 53.5 – 73.9 | 35.3 | 27.6 – 43.8 | 90.8 | 82.9 – 95.2 |
| Ceftazidime | 65.5 | 51.9 – 77.5 | 36.2 | 24.0 – 49.9 | 50.7 | 44.0 – 57.3 | 51.2 | 39.1 – 63.2 |
| Ciprofloxacin | 59.7 | 45.8 – 72.4 | 55.9 | 42.4 – 68.8 | 56.7 | 47.8 – 65.2 | 58.9 | 49.3 – 67.9 |
| Cefepime | 61.5 | 40.6 – 79.8 | 43.3 | 32.9 – 54.2 | 23.9 | 18.1 – 30.9 | 79.6 | 69.4 – 87.0 |
| Imipenem | 72.6 | 58.3 – 84.1 | 63.1 | 50.2 – 74.7 | 60.7 | 51.8 – 68.8 | 74.6 | 64.4 – 82.6 |
| Levofloxacin | 49.0 | 34.8 – 63.4 | 60.0 | 47.1 – 72.0 | 49.0 | 39.0 – 59.1 | 60.0 | 51.8 – 67.7 |
| Tobramycin | 70.9 | 59.6 – 80.6 | 54.1 | 36.9 – 70.5 | 76.7 | 69.3 – 82.8 | 46.5 | 35.6 – 57.8 |
| Piperacillin-tazobactam | 69.6 | 54.3 – 82.3 | 47.1 | 35.1 – 59.5 | 46.4 | 39.2 – 53.7 | 70.2 | 58.8 – 79.6 |

PPV: Positive predictive value; NPV: Negative predictive value; CI: Confidence interval.

**Table 8.** Accuracy, PLR, NLR, and DOR of the LS-AST method in comparison - manual method in *Pseudomonas aeruginosa* against different antibiotics.

|  | Accuracy | | PLR | | NLR | | DOR | |
| --- | --- | --- | --- | --- | --- | --- | --- | --- |
|  | % | [95% CI] |  | [95% CI] |  | [95% CI] |  | [95% CI] |
| Amikacin | 58.6 | 49.1 – 67.7 | 1.65 | 1.22 – 2.22 | 0.42 | 0.20 – 0.88 | 3.92 | 1.44 – 10.63 |
| Aztreonam | 66.4 | 57.0 – 74.9 | 2.09 | 1.46 – 2.99 | 0.39 | 0.19 – 0.79 | 5.36 | 1.94 – 14.84 |
| Ceftazidime | 50.9 | 41.4 – 60.3 | 1.03 | 0.78 – 1.34 | 0.95 | 0.58 – 1.56 | 1.08 | 0.50 – 2.31 |
| Ciprofloxacin | 57.8 | 48.2 – 66.9 | 1.35 | 0.95 – 1.94 | 0.72 | 0.49 – 1.06 | 1.88 | 0.90 – 3.92 |
| Cefepime | 47.4 | 38.1 – 56.9 | 1.09 | 0.76 – 1.55 | 0.89 | 0.52 – 1.52 | 1.22 | 0.50 – 2.99 |
| Imipenem | 67.2 | 57.9 – 75.7 | 1.96 | 1.37 – 2.82 | 0.44 | 0.27 – 0.71 | 4.51 | 2.04 – 10.00 |
| Levofloxacin | 55.2 | 45.7 – 64.4 | 1.23 | 0.81 – 1.84 | 0.85 | 0.61 – 1.19 | 1.44 | 0.69 – 3.02 |
| Tobramycin | 65.5 | 56.1 – 74.1 | 1.54 | 1.06 – 2.25 | 0.54 | 0.34 – 0.85 | 2.86 | 1.28 – 6.43 |
| Piperacillin-tazobactam | 56.0 | 46.5 – 65.2 | 1.32 | 0.98 – 1.76 | 0.65 | 0.39 – 1.07 | 2.04 | 0.93 – 4.47 |

PLR: Positive likelihood ratio; NLR: Negative likelihood ratio: DOR: Diagnostic odds ratio; CI: Confidence interval.

**Table 9.** Percentage of sensitivity, specificity, PPV, and NPV of the LS-AST method in comparison - manual method in *Proteus mirabilis* against different antibiotics.

|  | Sensitivity | | Specificity | | PPV | | NPV | |
| --- | --- | --- | --- | --- | --- | --- | --- | --- |
|  | % | [95% CI] | % | [95% CI] | % | [95% CI] | % | [95% CI] |
| Amikacin | 82.1 | 66.5 – 92.5 | 37.5 | 21.1 – 56.3 | 61.5 | 54.1 – 68.5 | 63.2 | 43.4 – 79.3 |
| Ampicillin | 43.8 | 26.4 – 62.3 | 76.9 | 60.7 – 88.9 | 60.9 | 43.7 – 75.7 | 62.5 | 54.0 – 70.3 |
| Gentamicin | 60.9 | 45.4 – 74.9 | 68 | 46.5 – 85.1 | 77.8 | 65.4 – 86.6 | 48.6 | 37.6 – 59.7 |
| Cefotaxime | 64.4 | 48.8 – 78.1 | 53.9 | 33.4 – 73.4 | 70.7 | 60.2 – 79.4 | 46.7 | 34.0 – 59.8 |
| Cefepime | 55 | 38.5 – 70.7 | 59.5 | 42.1 – 75.3 | 55 | 44.7 – 64.9 | 51.6 | 38.6 – 64.4 |
| Cefoxitin | 94.9 | 82.7 – 99.4 | 0.0 | 0.0 – 10.9 | 53.6 | 51.8 – 55.4 | NC | NC |
| Imipenem | 70.4 | 56.4 – 82.0 | 23.5 | 6.8 – 49.9 | 74.5 | 68.1 – 80.0 | 20.0 | 8.8 – 39.3 |
| Levofloxacin | 84 | 63.9 – 95.5 | 79 | 54.4 – 94.0 | 84 | 67.4 – 93.0 | 32.6 | 26.0 – 40.1 |
| Tobramycin | 54.9 | 40.3 – 68.9 | 75 | 50.9 – 91.3 | 84.9 | 71.6 – 92.6 | 39.5 | 30.5 – 49.2 |

PPV: Positive predictive value; NPV: Negative predictive value; CI: Confidence interval; NC: Not calculable.

**Table 10.** Accuracy, PLR, NLR, and DOR of the LS-AST method in comparison - manual method in *Proteus mirabilis* against different antibiotics.

|  | Accuracy | | PLR | | NLR | | DOR | |
| --- | --- | --- | --- | --- | --- | --- | --- | --- |
|  | % | [95% CI] |  | [95% CI] |  | [95% CI] |  | [95% CI] |
| Amikacin | 62.0 | 49.7 – 73.2 | 1.31 | 0.97 – 1.78 | 0.48 | 0.21 – 1.07 | 2.74 | 0.93 – 8.13 |
| Ampicillin | 62.0 | 49.7 – 73.2 | 1.90 | 0.95 – 3.80 | 0.73 | 0.51 – 1.04 | 2.59 | 0.93 – 7.2 |
| Gentamicin | 63.4 | 51.1 – 74.5 | 1.90 | 1.03 – 3.52 | 0.58 | 0.37 – 0.90 | 3.31 | 1.18 – 9.24 |
| Cefotaxime | 60.6 | 48.3 – 72.0 | 1.40 | 0.87 – 2.23 | 0.66 | 0.39 – 1.12 | 2.11 | 0.79 – 5.65 |
| Cefepime | 53.5 | 41.3 – 65.5 | 1.12 | 0.74 – 1.70 | 0.86 | 0.51 – 1.46 | 1.3 | 0.51 – 3.34 |
| Cefoxitin | 52.1 | 39.9 – 64.1 | 0.95 | 0.88 – 1.02 | NC | NC | NC | NC |
| Imipenem | 59.2 | 46.8 – 70.7 | 0.92 | 0.67 – 1.26 | 1.26 | 0.49 – 3.26 | 0.73 | 0.21 – 2.59 |
| Levofloxacin | 50.7 | 38.6 – 62.8 | 1.92 | 0.76 – 4.87 | 0.76 | 0.55 – 1.04 | 2.54 | 0.74 – 8.73 |
| Tobramycin | 60.6 | 48.3 – 72.0 | 2.20 | 0.99 – 4.88 | 0.60 | 0.41 – 0.89 | 3.65 | 1.15 – 11.56 |

PLR: Positive likelihood ratio; NLR: Negative likelihood ratio: DOR: Diagnostic odds ratio; CI: Confidence interval; NC: Not calculable.
